# Vitamin D Insufficiency in Arabs and South Asians Positively Associates with Polymorphisms in GC and CYP2R1 Genes

**DOI:** 10.1371/journal.pone.0113102

**Published:** 2014-11-18

**Authors:** Naser Elkum, Fadi Alkayal, Fiona Noronha, Maisa M. Ali, Motasem Melhem, Monira Al-Arouj, Abdullah Bennakhi, Kazem Behbehani, Osama Alsmadi, Jehad Abubaker

**Affiliations:** 1 Department of Biostatistics & Epidemiology, Dasman Diabetes Institute, Kuwait City, Kuwait; 2 Genetics & Genomics Unit, Dasman Diabetes Institute, Kuwait City, Kuwait; 3 Biochemistry & Molecular Biology Unit, Dasman Diabetes Institute, Kuwait City, Kuwait; 4 Clinical Services, Dasman Diabetes Institute, Kuwait City, Kuwait; 5 Clinical Epidemiology, Sidra Medical and Research Centre, Doha, Qatar; National Cancer Center, Japan

## Abstract

**Background:**

A number of genetic studies have reported an association between vitamin D related genes such as group-specific component gene (GC), Cytochrome P450, family 2, subfamily R, polypeptide 1 (CYP2R1) and 7-dehydrocholesterol reductase/nicotinamide-adenine dinucleotide synthetase 1 (DHCR7/NADSYN1) and serum levels of the active form of Vitamin D, 25 (OH) D among African Americans, Caucasians, and Chinese. Little is known about how genetic variations associate with, or contribute to, 25(OH)D levels in Arabs populations.

**Methods:**

Allele frequencies of 18 SNPs derived from CYP2R1, GC, and DHCR7/NADSYN1 genes in 1549 individuals (Arabs, South Asians, and Southeast Asians living in Kuwait) were determined using real time genotyping assays. Serum levels of 25(OH)D were measured using chemiluminescence immunoassay.

**Results:**

GC gene polymorphisms (rs17467825, rs3755967, rs2282679, rs7041 and rs2298850) were found to be associated with 25(OH)D serum levels in Arabs and South Asians. Two of the CYP2R1 SNPs (rs10500804 and rs12794714) and one of GC SNPs (rs1155563) were found to be significantly associated with 25(OH)D serum levels only in people of Arab origin. Across all three ethnicities none of the SNPs of DHCR7/NADSYN1 were associated with serum 25(OH)D levels and none of the 18 SNPs were significantly associated with serum 25(OH)D levels in people from South East Asia.

**Conclusion:**

Our data show for the first time significant association between the GC (rs2282679 and rs7041), CYP2R1 (rs10741657) SNPs and 25(OH)D levels. This supports their roles in vitamin D Insufficiency in Arab and South Asian populations respectively. Interestingly, two of the CYP2R1 SNPs (rs10500804 and rs12794714) and one GC SNP (rs1155563) were found to correlate with vitamin D in Arab population exclusively signifying their importance in this population.

## Introduction

Vitamin D deficiency is a common public health problem worldwide. It is associated with many medical outcomes, including osteoporosis [1], type 1 diabetes [2], cardiovascular diseases [3], asthma [4], and cancer [5]. Vitamin D plays a major role in calcium and phosphate homeostasis, both of which are essential in the mineralization of bone, muscle contraction, nerve conduction, and general cellular function in all cells of the body. Its active form, 1,25-dihydroxyvitamin D [1,25(OH)2D], control the expression of a vitamin D-dependent genes that code for calcium-transporting and bone matrix proteins [6]. The best indicator of vitamin D status is the serum concentration of its main circulating metabolite, 25-hydroxyvitamin D [25(OH)D].

Factors that can potentially affect vitamin D status are dietary intake and exposure to ultraviolet-B (UVB) sunlight. Sunlight exposure catalyzes vitamin D photochemical synthesis from a cholesterol-like precursor in the skin, which is by far the most important source of vitamin D [7], [8] and therefore limited exposure to sunlight is thought to be a key factor in vitamin D deficiency [9], [10]. In Arabian Gulf countries where there is plentiful sunlight throughout the year, vitamin D could be expected to be adequate. Studies in Saudi Arabia and United Arab Emirates have nevertheless highlighted a high prevalence of vitamin D deficiency in the local populations [10], [11].

A study of a multi-ethnic population in the United Arab Emirates (UAE) [11] found serum 25(OH)D levels to be deficient in the overall population but sufficient among the Europeans contingent living in the same environment. Other studies have also reported wide ethnic differences. One study found a vitamin D insufficiency rate in Moroccans of 91% [12]. In the USA, Arab-American women living in southeast Detroit have been found to have dangerously low vitamin D levels [13]. These finding and others suggest potential genetic influences that predispose people of Arab backgrounds to vitamin D deficiency [14], [15].

In this study, we look at which genetic variants underlie 25(OH)D status and how single nucleotide polymorphisms (SNPs) in key genes may be influencing vitamin D status. For example, SNPs in enzymes required for the production or secretion of 25(OH)D and 1,25-dihydroxyvitamin D [1,25(OH)D] could influence serum concentrations and polymorphism (e.g. if it results in more or less efficient enzyme, receptor, or binding protein), could either increase or decrease the concentration of 25(OH)D in sera.

Recent genetic studies have associated vitamin D deficiency with several candidate genes including Cytochrome P450, family 2, R, (CYP2R1), the group-specific component gene (GC) and 7-dehydrocholesterol reductase/NAD synthetase 1 (DHCR7/NADSYN1) [16]–[19]. These genes are involved in cholesterol synthesis (DHCR7/NADSYN1), hydroxylation (CYP2R1), and vitamin D transport (GC). The association between the polymorphisms of these genes and 25(OH)D has been previously studied in populations of European descent [18], [20], [21], African Americans [22], [23], and Chinese [19], [24]. Little is known about these associations in Arab populations. This study set out to (a) measure levels of 25(OH)D in Arabs, South Asians, and Southeast Asians; (b) to estimate the allelic frequencies of 18 SNPs from CYP2R1, GC, and DHCR7/NADSYN1 genes; and to investigate the relationship between these genetic polymorphisms and the level of 25(OH)D in Arab and Asian populations. In this study, we report for the first time the significant association between SNPs from GC (rs2282679 and rs7041), CYP2R1 (rs10741657) genes and 25(OH)D levels which clearly support their roles in vitamin D Insufficiency in Arab and South Asian populations. The lack of association between DHCR7/NADSYN1 SNPs and 25(OH)D levels minimize their role in controlling vitamin D levels in all three ethnic groups. Moreover, the fact that two of the CYP2R1 SNPs (rs10500804 and rs12794714) and one GC SNP (rs1155563) were found to associate with Vitamin D levels exclusively in Arabs signify their role in vitamin D insufficiency within this population.

## Materials and Methods

### Study population

A cross-sectional population-based survey was conducted with a random representative sample of adults (≥18 years) from multi-ethnic origin across the six governorates (strata) of the State of Kuwait. A full description of the study population, design and data collection of this study have outlined previously [25]. Briefly, a stratified random sampling technique was used for the selection of participants from the computerized register of the Public Authority of Civil Information. This survey was carried out between June 2011 and August 2012. The study conformed to the principles outlined in the Declaration of Helsinki and was approved by the Scientific Advisory Council and Ethical Review Committee at the Dasman Diabetes Institute (DDI) IRB # 1– Biomedical. An informed written consent was obtained from all participants before their enrolment in the study.

### Measurement of vitamin D levels

Serum 25(OH)D levels were measured by chemiluminescent competitive immunoassay (CLIA) using a DiaSorin LIAISON analyzer (DiaSorin Inc, MN, USA) and following company instruction. In brief, 25 OH Vitamin D was dissociated from its binding protein and bound to a specific antibody on the solid phase. Then the tracer (vitamin D linked to an isoluminol derivative) was added. Next, the unbound material was removed with a wash cycle. Finally, the starter reagents were added to initiate a flash chemiluminescent reaction. The light signal was measured by a photomultiplier as relative light units (RLU) and was inversely proportional to the concentration of 25(OH)D in the samples. The intra-assay coefficients of variations (CVs) were 5.5% and 4.0% at 10 and 25 ng/mL, respectively. The inter-assay CVs were 8% and 6% at 15 and 40 ng/mL, respectively. Body Mass Index (BMI) was calculated, using the standard BMI formula, as body weight (in kilograms) divided by height (in meters) squared. This study was conducted on adult male and female subjects comprising of Normal (BMI = 20–24.9 kg/m^2^), overweight (BMI = 25–29.9 kg/m^2^) and obese (BMI≥30 kg/m^2^) individuals. Other measurements such age, weight, height, gender and ethnicity were also obtained.

### DNA collection, SNP selection, and genotyping

Blood samples were taken from consenting participants in accordance with DDI IRB approved consent form in 4 ml tubes containing EDTA anticoagulant. Gentra Puregene kit (Qiagen, Valencia, CA, USA) was used to extract DNA as per manufacturer’s protocols. DNA was quantified, with a requirement that the A260/A280 ratio is in the range of 1.8–2.1, using Epoch Microplate Spectrophotometer. DNA stock aliquots were diluted to a concentration of 50 ng/µl and were frozen until needed for use in PCR assays.

We selected three candidate genes containing 18 SNPs (6 SNPs from each gene) that have been shown in previous GWAS reports [17], [18] to have an association with vitamin D level, and are known to have a biological impact in vitamin D metabolism. The genes and SNPs included GC (rs17467825, rs2282679, rs3755967, rs2298850, rs7041, rs1155563), CYP2R1 (rs7116978, rs1993116, rs10500804, 4s12794714, rs10741657, rs206793), and DHCR7/NADSYN1 (rs7944926, rs12785878, rs4944957, rs12800438, rs3794060, and rs3829251).

We employed ready-to-use, manufacturer-validated, pre-designed allele discriminating TaqMan single nucleotide polymorphisms (SNP) assays. PCR amplification reactions were each carried out in clear optical 96-well plates on the Applied Biosystems (ABI) 7500 Real Time PCR system. Each reaction was performed in 20 µl volume, containing 20 ng of DNA template, 1X TaqMan pre-designed SNP assay master mix (Applied Biosystems, Carlsbad, CA, United States), and 1X HOT FIREPol EvaGreen qPCR master mix plus containing ROX reference dye (Solis BioDyne, Tartu, Estonia). Amplification cycling reactions were carried out under the following conditions: an initial incubation step at 95°C for 10 minutes followed by 35 cycles at 95°C for 1 minute, 58°C for 45 seconds, and 72°C for 45 seconds, then a final incubation step at 72°C for 7 minutes. An endpoint “plate read” was then performed using the ABI Sequence Detection System (SDS) Software. The Software detects fluorescence released from the bi-allelic TaqMan probes and plots fluorescence (Rn) signals (FAM & VIC) and generates the genotype calls. In every run, two previously validated DNA controls per genotype were included, in addition to two water (no template) negative controls.

### Statistical analysis

We compared the baseline characteristics of the participants using analysis of variance tests (ANOVA) for continuous variables. Categorical variables were analyzed using the chi-square test. Mean serum 25(OH)D values were estimated within each group of homozygous referent (HR), heterozygous (HET), and homozygous variant (HV) genotypes for each SNP. The serum 25(OH)D levels were adjusted by age, gender and BMI using analysis of covariance (ANCOVA) approach. A *P*-value<0.05 was considered to be statistically significant. All analyses were performed using SAS (version 9.2; SAS Institute, Cary, NC). Genotype frequencies were determined according to the Hardy-Weinberg equilibrium (HWE).

## Results

### Clinical and biochemical characteristics of the participants

The study population (1549 in total) comprised subjects mainly of Arab and Asian background; 907 Arabs, 489 South Asians and 153 Southeast Asians. The descriptive characteristics of all participants are summarized in Table 1. There were substantial ethnic differences in BMI, age, and gender (p<0.0001); Arabs had a significantly higher mean BMI and tended to be older than Asians. Southeast Asians had higher mean levels of 25(OH)D compared to Arabs and South Asians.

**Table 1:** Characteristics of participants by ethnicity.

Factors	Ethnicities			*P-value* trend
	Arabs	South-East Asia	South Asia	
	n (%)	n (%)	n (%)	
Age (years)				
20–39	316 (20.3)	90 (5.8)	186 (11.9)	<0.0001
40–60	497 (31.9)	59 (3.8)	276 (17.8)	<0.0001
>60	98 (6.3)	5 (0.32)	28 (1.8)	<0.0001
Gender				
Female	270 (17.4)	104 (6.7)	104 (6.7)	<0.0001
Male	641 (41.2)	50 (3.2)	386 (24.8)	<0.0001
BMI				
Normal (18.5–24.9)	107 (6.9)	62 (3.9)	143 (9.2)	<0.0001
Overweight (25–29.9)	296 (19.0)	65 (4.2)	225 (14.5)	<0.0001
Obese (≥30)	508 (32.7)	27 (1.7)	122 (7.9)	<0.0001
*25 OHD (ng/l)	13.5±0.34	17.8±0.82	13.3±0.46	<0.0001

BMI, body mass index; *Data are presented as mean ± SD.

### Distribution of allele frequencies in different populations

**Figure 1:**
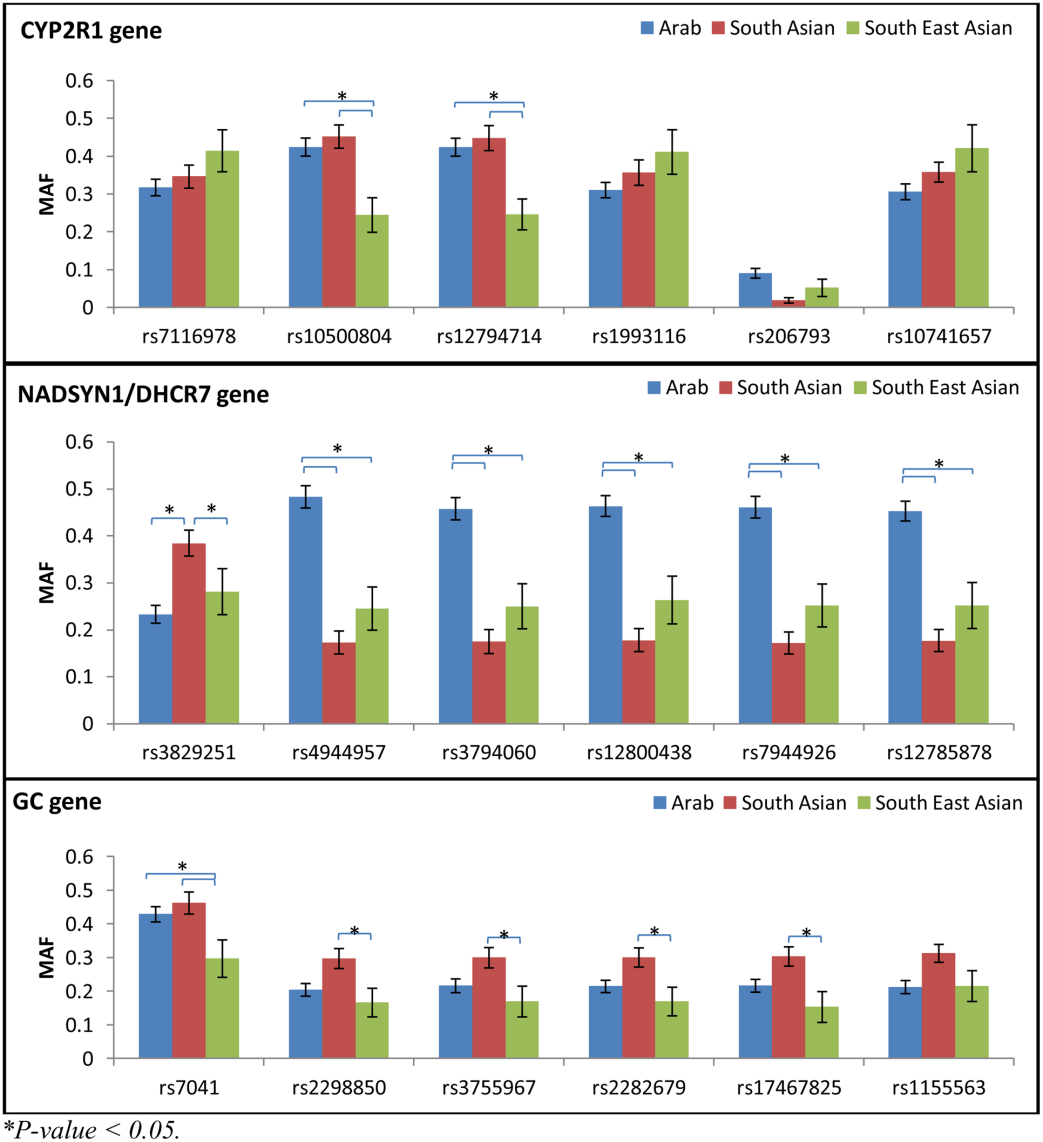
Minor Allele Frequency of SNPs from CYP2R1, DHCR7 & GC genes between Arabs, South Asian and South East Asian ethnicities. The standard deviation was estimated using the.

Significant differences were found in the allele frequencies across all three study populations. The distribution of minor alleles frequencies of each SNP in the Arab, South Asia, and Southeast Asia populations are shown in Figure 1. Minor alleles (frequency below 0.20) were less common in the Arab population (5.3%) than in the South Asian (31.6%) and Southeast Asian (26.4%) populations. Hardy-Weinberg equilibrium (HWE) was not met for some SNPs across all three-populations (P<0.05). Three CYP2R1 SNPs in the Arab population and four GC SNPs in the Southeast Asian population had HWE<0.05 (Table 2).

**Table 2:** Allele frequencies of 18 Vitamin D associated SNPs by ethnicity.

Gene & SNP	Region	Allele	MAF		Arab n = 907			South Asian n = 489			South East Asian n = 153		
			All^a^	All^b^	MAF	HET	HWE	MAF	HET	HWE	MAF	HET	HWE
***CYP2R1***													
rs7116978	Ch 11	C/T	0.36	0.33	0.32	0.40	**0.0268**	0.35	0.43	0.2123	0.42	0.44	0.2248
rs10500804	Ch 11	T/G	0.43	0.42	0.42	0.45	**0.0164**	0.45	0.49	0.7610	0.24	0.39	0.6027
rs12794714	Ch 11	G/A	0.43	0.42	0.42	0.45	**0.0169**	0.45	0.48	0.5556	0.25	0.39	0.5849
rs1993116	Ch 11	G/A	0.40	0.33	0.31	0.42	0.6667	0.36	0.44	0.2498	0.41	0.44	0.3073
rs206793	Ch 11	T/C	0.40	0.07	0.09	0.16	0.0710	0.02	0.04	0.6613	0.05	0.10	0.4950
rs10741657	Ch 11	G/A	0.40	0.33	0.31	0.42	0.5537	0.36	0.43	0.1540	0.42	0.44	0.2067
***DHCR7/NADSYN1***													
rs3829251	Ch 11	G/A	0.24	0.28	0.23	0.36	0.9608	0.38	0.47	0.3126	0.28	0.41	0.9729
rs4944957	Ch 11	A/G	0.23	0.37	0.48	0.49	0.6672	0.17	0.40	0.4225	0.24	0.37	0.9334
rs3794060	Ch 11	C/T	0.23	0.35	0.46	0.49	0.6768	0.18	0.39	0.1186	0.25	0.38	0.8288
rs12800438	Ch 11	G/A	0.23	0.36	0.46	0.49	0.6117	0.18	0.39	0.1430	0.26	0.37	0.5376
rs7944926	Ch 11	A/G	0.23	0.35	0.46	0.49	0.6534	0.17	0.40	0.2068	0.25	0.39	0.7677
rs12785878	Ch 11	G/T	0.23	0.35	0.45	0.49	0.5725	0.18	0.42	0.5105	0.25	0.40	0.4687
***GC***													
rs7041	Ch 4	**C**/A	0.44	0.47	0.43	0.47	0.1302	0.46	0.47	0.3126	0.30	0.39	0.3394
rs2298850	Ch 4	G/C	0.28	0.23	0.20	0.32	0.6739	0.30	0.40	0.4225	0.17	0.20	**0.0008**
rs3755967	Ch 4	C/T	0.29	0.24	0.22	0.33	0.4866	0.30	0.39	0.1186	0.17	0.21	**0.0014**
rs2282679	Ch 4	T/G	0.29	0.24	0.21	0.33	0.6582	0.30	0.39	0.1430	0.17	0.21	**0.0014**
rs17467825	Ch 4	A/G	0.29	0.24	0.22	0.33	0.6172	0.30	0.40	0.2068	0.15	0.19	**0.0008**
RS1155563	Ch 4	T/C	0.30	0.25	0.21	0.32	0.2397	0.31	0.42	0.5105	0.22	0.31	0.3683

Allele major allele/minor allele; MAF minor allele frequency; HET heterozygosity; HWE *P*-values for Hardy-Weinberg Equilibrium test.

Bold numbers represent significant *P*-values.

a Based on GWAS by Wang, et al. 2010;

b based on our study.

### Association between genotypes and 25(OH) D serum concentrations

SNP genotype and mean serum 25(OH)D levels categorized by genotype are presented in Table 3. Only two CYP2R1 SNPs (rs10500804 and rs12794714) in Arab and one SNP (rs10741657) in South Asian populations were positively associated with vitamin D. No association was observed between the CYP2R1 gene and vitamin D in the South East Asian population. As for the GC gene, all the SNPs were significantly associated with serum 25(OH)D levels in the Arab population but not in the South East Asia population. In South Asian group, five SNPs in GC the gene (rs17467825, rs2282679, rs3755967, rs2298850, and rs7041) were significantly associated with vitamin D levels. SNPs in DHCR7/NADSYN1 gene showed no significant association with serum 25(OH)D in any of the three study populations. Table S1 shows unadjusted analysis of the association between genotypes and 25(OH)D serum levels.

**Table 3:** Single nucleotide polymorphisms in GC, CYP2R1, DHCR7/NADSYN1, and their association with serum 25(OH)D among Arabs, South Asian, Southeast Asian participants.

Gene & SNP	Region	HR, HET, HV	Arab n = 907 25(OH)D concentrations				South Asian n = 489 25(OH)D concentrations				South East Asian n = 153 25(OH)D concentrations			
			HR	HET	HV	*P*-values	HR	HET	HV	*P*-values	HR	HET	HV	*P*-values
***CYP2R1***														
rs7116978	Ch 11	CC, CT, TT	13.5	14.2	14.4	0.5747	13.7	14.4	16.2	0.1068	16.7	18.3	16.1	0.3397
rs1993116	Ch 11	GG, AG, AA	13.3	14.5	14.1	0.2736	13.4	14.6	16.2	0.0555	17.1	17.7	16.8	0.8459
rs10500804	Ch 11	TT, TG, GG	14.4	14.3	12.0	**0.0379**	14.5	14.7	13.3	0.3770	17.8	16.7	16.4	0.6780
rs12794714	Ch 11	GG, AG, AA	14.4	14.3	12.0	**0.0402**	14.5	14.8	13.1	0.2790	17.8	16.7	16.4	0.7009
rs10741657	Ch 11	GG, AG, AA	13.3	14.5	14.1	0.2873	13.4	14.6	16.2	**0.0437**	16.9	17.9	16.7	0.7224
rs206793	Ch 11	TT, CT, CC	13.9	13.7	9.6	0.3804	14.3	13.8	-	0.7915	17.4	16.4	-	0.6456
***DHCR7/NADSYN1***														
rs7944926	Ch 11	AA, AG, GG	13.2	14.1	14.2	0.5300	13.9	15.5	11.9	0.1096	16.4	18.1	21.9	0.1111
rs12785878	Ch 11	GG, GT, TT	13.2	14.2	14.0	0.5164	14.0	15.4	12.4	0.1660	16.2	18.2	22.2	0.0736
rs4944957	Ch 11	AA, AG, GG	13.0	14.1	14.4	0.3336	14.0	15.5	11.9	0.1234	16.6	17.8	21.9	0.1577
rs12800438	Ch 11	GG, AG, AA	13.3	14.1	14.2	0.6303	14.1	15.2	12.7	0.2761	16.3	18.9	16.0	0.1371
rs3794060	Ch 11	CC, CT, TT	13.3	14.1	14.3	0.5396	14.0	15.5	12.3	0.1364	16.4	18.1	21.9	0.1135
rs3829251	Ch 11	GG, AG, AA	14.4	12.9	14.1	0.1833	13.6	14.9	13.9	0.2599	18.1	16.8	15.0	0.3578
***GC***														
rs17467825	Ch 4	AA, AG, GG	14.4	13.4	10.3	**0.0165**	15.8	12.9	13.0	**0.0013**	17.7	17.4	12.8	0.2127
rs2282679	Ch 4	TT, GT, GG	14.4	13.3	10.4	**0.0377**	15.8	12.8	13.0	**0.0007**	17.8	17.0	12.8	0.1557
rs3755967	Ch 4	CC, CT, TT	14.4	13.3	10.5	**0.0368**	15.8	12.8	13.0	**0.0007**	17.8	17.0	12.8	0.1557
rs2298850	Ch 4	GG, GC, CC	14.4	13.3	10.3	**0.0374**	15.5	13.2	13.0	**0.0103**	17.9	16.7	13.4	0.2037
rs7041	Ch 4	CC, AC, AA	14.5	14.3	11.7	**0.0110**	15.8	14.4	12.3	**0.0072**	17.3	18.1	16.6	0.5594
rs1155563	Ch 4	TT, TC, CC	14.4	13.3	10.4	**0.0289**	14.8	14.2	12.8	0.3120	18.1	16.2	14.7	0.2541

HR: Homozygous referent, HET: Heterozygous, HV: Homozygous variant. Blue color indicates HV.

*P*-value for the association between the SNP and 25(OH)D levels from ethnic-stratified ANCOVA models adjusted for sex, age and BMI.

## Discussion

We conducted this research because different studies have recently shown ethnic differences in the allele frequency of “vitamin D associated SNPs” [22], [26], [27], but little information is available about the frequency of vitamin D SNPs in Arab and Asian populations. Our data show that the minor allele frequency (MAF<0.20) was higher in Asian populations (South Asians 31.6% and South East Asians 26.3%) compared to Arabs (5.3%); this can be attributed to an ascertainment bias as the SNPs examined were from studies representing European and African ethnicities. In the GC gene, MAFs for South Asians were significantly higher (P<0.05) than Arabs and South East Asians (Figure 1), whereas South East Asians have the lowest frequencies among all the SNPs. The Arab population has shown distinctive significant differences in the allele frequency of the NADSYN1/DHCR7 gene compared to other ethnic groups. Arabs showed the highest MAF among all the SNPs of the NADSYN1/DHCR7 gene; only the rs3829251 SNP demonstrated higher frequencies in South Asians. Furthermore, analyses showed no significant differences in MAFs between Arab and South Asians populations across the CYP2R1 SNPs (rs206793 was an exception). These differences in allele frequencies are of significant importance in the design of association studies and selecting candidate vitamin D SNPs to investigate.

We were also interested in exploring the relationships between SNPs from the key Vitamin D genes (GC, CYP2R1 and DHCR7/NADSYN1) and 25(OH)D levels. The GC and CYP2R1 genes were found to be significantly associated with 25(OH)D levels. The GC gene encodes the vitamin D binding protein (DBP) that is the key transporter of vitamin D and its metabolites (including 25(OH)D and 1,25(OH)_2_D) in the circulation [28]. Recent studies have reported an association between SNPs in this gene and 25(OH)D concentrations [22], [23], [29], [30]. Furthermore, other recent studies involving African Americans and Europeans have reported a significant association between SNP rs2282679 of the GC gene and vitamin D insufficiency [17], [23]. It is notable that we found a strong association between SNP rs2282679 and 25(OH)D in Arabs and South Asians but not in South East Asians. Similar to the aforementioned studies on different ethnicities [24], rs7041 polymorphism was also associated with 25(OH)D in Arab and South Asian populations, but this was not the case with South East Asians. We also observed that polymorphisms rs17467825, rs3755967, and rs2298850 in the GC gene were associated with serum 25(OH)D levels in Arab and South Asian populations. Interestingly, rs1155563 SNP associated significantly with vitamin D level in Arabs, which suggests a possible involvement in Vitamin D secretion and transportation in this group. A previous study has found that the SNP rs1155563 was associated with vitamin D levels in men of non-Hispanic white background [30]. The significant associations between these GC SNPs and 25(OH)D levels further support the importance of the GC gene in Vitamin D insufficiency.

CYP2R1 is a microsomal vitamin D hydroxylase that hydroxylates vitamin D at the 25-C position for 25(OH)D synthesis (calcidiol) in the liver [31]. Subsequently, calcidiol is converted to calcitriol, the active form of vitamin D3 that binds to the vitamin D receptor (VDR) which arbitrates the majority of vitamin D physiological actions. Previous research has shown the gene CYP2R1 to be associated with several vitamin D related diseases such as type 1 diabetes [21], and in this study we found that rs10741657 SNP, which is a coding SNP that can change the activity of the CYP2R1 enzyme and subsequently cause a relative lack of 25(OH)D, is significantly associate with vitamin D, but only in South Asians [31]. We also found two of the CYP2R1 SNPs (rs10500804 and rs12794714) to associate significantly with Vitamin D level in Arabs signifying their possible roles in vitamin D insufficiency in this population.

Gene DHCR7/NADSYN1 encodes the enzyme 7-dehydrocholesterol (7DHC) reductase, which catalyzes the production of cholesterol from 7 DHC, thereby removing the key substrate necessary for the vitamin D synthesis [18]. Recently, Zhang et al., 2012 have linked some of the DHCR7/NADSYN1 SNPs (rs3829251, rs12785878) to decreased serum 25(OH)D levels in northeastern Han Chinese children [24], while Cooper et al., 2011 has associated rs12785878 T allele carriers with vitamin D deficiency and type 1 diabetes [32]. In contrast, we found that none of the six variant genotypes of DHCR7/NADSYN1 was associated with serum 25(OH)D levels in any of the three population groups that were studied, suggesting minimal involvement of these SNPs or the DHCR7/NADSYN1 gene in mediating vitamin D insufficiency in these populations. However, it is worth noting that in our study the female participant from Arab and South Asian origins were underrepresented which might contribute to the lack of association between DHCR7/NADSYN1 gene and serum 25(OH)D levels. Interestingly, none of the 18 SNPs in this study associated significantly with serum 25(OH)D levels in South East Asians, minimizing the roles of these genes in mediating Vitamin D insufficiency and thus opening the door for the possible involvement of other genes, less common SNPs and/or predisposing factors for Vitamin D insufficiency.

## Conclusion

Our study is one of the first to look at genetic determinant of vitamin D levels in Arabs and describes allele frequencies of 18 SNPs localized to three genes related to vitamin D deficiency in Arabs, South Asians and South East Asians. The significant associations between the GC (rs2282679 and rs7041), CYP2R1 (rs10741657) SNPs and 25(OH)D levels clearly support the idea of a role in vitamin D insufficiency in Arab and South Asian populations. The fact that none of the SNPs of the DHCR7/NADSYN1 gene associate with vitamin D levels in the three populations suggests minimized roles in controlling vitamin D release. The finding that GC SNP (rs1155563) and CYP2R1 SNPs (rs10500804 and rs12794714) associated exclusively with vitamin D level in Arabs, suggests the need for further study and possibly sheds light on their mechanism in the context of vitamin D insufficiency. Finally, the lack of association between the selected SNPs and vitamin D levels in South East Asians calls for larger population-based studies that include more genes linked to vitamin D and/or explore the less common SNPs within the existing genes.

## Supporting Information

Table S1 Single nucleotide polymorphisms in GC, CYP2R1, DHCR7/NADSYN1, and their association with serum 25(OH)D among Arabs, South Asian, Southeast Asian participants.
(DOCX)
