# Supplementary material for: Vitamin D Insufficiency in Arabs and South Asians Positively Associates with Polymorphisms in GC and CYP2R1 Genes
Source: PLoS One. 2014 Nov 18;9(11):e113102. doi: 10.1371/journal.pone.0113102 (PMC4236149; doi:10.1371/journal.pone.0113102)
Supplement: Table S1 — Single nucleotide polymorphisms in GC, CYP2R1, DHCR7/NADSYN1, and their association with serum 25(OH)D among Arabs, South Asian, Southeast Asian participants. (DOCX) [file pone.0113102.s001.docx]

**Table S1:** Single nucleotide polymorphisms in GC, CYP2R1, DHCR7/NADSYN1, and their association with serum 25(OH)D among Arabs, South Asian, Southeast Asian participants.

| **Gene & SNP** | **Region** | **HR, HET, HV** | **Arab**  **n = 907** | | | | **South Asian**  **n = 489** | | | | | | | | **South East Asian**  **n =153** | | | |
| --- | --- | --- | --- | --- | --- | --- | --- | --- | --- | --- | --- | --- | --- | --- | --- | --- | --- | --- |
|  |  |  | **HR** | **HET** | **HV** | ***P*-values** | **HR** | | **HET** | | | **HV** | | ***P*-values** | **HR** | **HET** | **HV** | ***P*-values** |
| ***CYP2R1*** | | | | | | | | | | | | | | | | | | |
| rs7116978 | Ch 11 | CC, CT, TT | 13.1 | 13.8 | 13.6 | 0.6429 | 12.7 | 13.5 | | | 14.9 | | 0.1792 | | 16.9 | 19.2 | 16.4 | 0.1621 |
| rs1993116 | Ch 11 | GG, AG, AA | 12.9 | 14.2 | 13.4 | 0.2459 | 12.3 | 13.7 | | | 14.9 | | 0.0656 | | 17.3 | 18.7 | 16.7 | 0.4818 |
| rs10500804 | Ch 11 | TT, TG, GG | 13.8 | 14.0 | 11.5 | 0.0310 | 13.2 | 13.8 | | | 12.3 | | 0.3513 | | 18.4 | 16.9 | 18.0 | 0.6037 |
| rs12794714 | Ch 11 | GG, AG, AA | 13.8 | 14.0 | 11.5 | 0.0316 | 13.2 | 13.9 | | | 12.1 | | 0.2413 | | 18.3 | 16.9 | 18.0 | 0.6394 |
| rs10741657 | Ch 11 | GG, AG, AA | 12.9 | 14.1 | 13.3 | 0.2686 | 12.3 | 13.9 | | | 14.9 | | 0.0517 | | 17.2 | 18.8 | 16.7 | 0.3992 |
| rs206793 | Ch 11 | TT, CT, CC | 13.6 | 13.2 | 9.7 | 0.4414 | 13.3 | 12.7 | | |  | | 0.7505 | | 17.9 | 16.5 |  | 0.4903 |
| ***DHCR7/NADSYN1*** | | | | | | | | | | | | | | | | | | |
| rs7944926 | Ch 11 | AA, AG, GG | 12.7 | 13.8 | 13.7 | 0.4036 | 12.9 | | 14.5 | 11.3 | | | 0.1570 | | 16.9 | 18.5 | 22.4 | 0.1206 |
| rs12785878 | Ch 11 | GG, GT, TT | 12.7 | 13.8 | 13.6 | 0.4207 | 12.9 | | 14.4 | 11.9 | | | 0.2460 | | 16.8 | 18.6 | 22.8 | 0.0919 |
| rs4944957 | Ch 11 | AA, AG, GG | 12.5 | 13.8 | 13.9 | 0.2617 | 13.0 | | 14.4 | 11.3 | | | 0.1761 | | 17.1 | 18.2 | 22.4 | 0.1623 |
| rs12800438 | Ch 11 | GG, AG, AA | 12.8 | 13.7 | 13.7 | 0.5335 | 13.1 | | 14.2 | 11.7 | | | 0.3214 | | 16.9 | 19.2 | 16.8 | 0.2580 |
| rs3794060 | Ch 11 | CC, CT, TT | 12.7 | 13.7 | 13.8 | 0.4363 | 12.9 | | 14.4 | 11.5 | | | 0.1818 | | 16.9 | 18.5 | 22.4 | 0.1186 |
| rs3829251 | Ch 11 | GG, AG, AA | 14.0 | 12.5 | 13.6 | 0.1325 | 12.6 | | 13.9 | 13.2 | | | 0.2809 | | 18.8 | 16.9 | 15.4 | 0.2463 |
| ***GC*** | | | | | | | | | | | | | | | | | | |
| rs17467825 | Ch 4 | AA, AG, GG | 14.0 | 12.8 | 10.2 | 0.0422 | 14.6 | | 11.9 | 12.2 | | | 0.0037 | | 18.2 | 17.7 | 12.9 | 0.1885 |
| rs2282679 | Ch 4 | TT, GT, GG | 14.0 | 12.8 | 10.4 | 0.0485 | 14.7 | | 11.8 | 12.2 | | | 0.0020 | | 18.5 | 17.2 | 12.7 | 0.0942 |
| rs3755967 | Ch 4 | CC, CT, TT | 14.0 | 12.8 | 10.5 | 0.0495 | 14.7 | | 11.8 | 12.2 | | | 0.0019 | | 18.5 | 17.2 | 12.7 | 0.0942 |
| rs2298850 | Ch 4 | GG, GC, CC | 14.0 | 12.8 | 10.2 | 0.0477 | 14.4 | | 12.2 | 12.5 | | | 0.0247 | | 18.5 | 16.8 | 13.2 | 0.1169 |
| rs7041 | Ch 4 | CC, AC, AA | 14.2 | 13.9 | 11.2 | 0.0075 | 14.6 | | 13.3 | 11.6 | | | 0.0250 | | 18.1 | 18.6 | 17.2 | 0.6121 |
| rs1155563 | Ch 4 | TT, TC, CC | 14.1 | 12.8 | 10.2 | 0.0312 | 13.7 | | 13.2 | 12.0 | | | 0.4462 | | 18.7 | 16.4 | 15.3 | 0.1788 |

HR: Homozygous referent, HET: Heterozygous, HV: Homozygous variant..

*P*-value for the association between the SNP and 25(OH)D levels from ethnic-stratified ANOVA models.
